# Human Ghrelin Mitigates Intestinal Injury and Mortality after Whole Body Irradiation in Rats

**DOI:** 10.1371/journal.pone.0118213

**Published:** 2015-02-11

**Authors:** Zhimin Wang, Weng Lang Yang, Asha Jacob, Monowar Aziz, Ping Wang

**Affiliations:** 1 Center for Translational Research, The Feinstein Institute for Medical Research, Manhasset, NY, United States of America; 2 TheraSource LLC, Manhasset, NY, United States of America; National Institute of Agronomic Research, FRANCE

## Abstract

Widespread use of ionizing radiation has led to the realization of the danger associated with radiation exposure. Although studies in radiation countermeasures were initiated a half century ago, an effective therapy for a radiomitigator has not been identified. Ghrelin is a gastrointestinal hormone, and administration of ghrelin is protective in animal models of injuries including radiation combined injury. To test whether ghrelin can be protective in whole body irradiaton (WBI) alone, male Sprague Dawley (SD) rats were treated with human ghrelin (20 nmol/rat) daily for 6 days starting at either 24 h or 48 h after 10 Gray (Gy) WBI and survival outcome was examined. The 10 Gy WBI produced a LD^70/30^ model in SD rats (30% survival in 30 days). The survival rate in rats treated with ghrelin starting at 24 h was significantly improved to 63% and when treatment was initiated at 48 h, the survival remained at 61%. At 7 days post WBI, plasma ghrelin was significantly reduced from the control value. Ghrelin treatment starting at 24 h after WBI daily for 6 days improved histological appearance of the intestine, reduced gut permeability, serum endotoxin levels and bacterial translocation to the liver by 38%, 42% and 61%, respectively at day 7 post WBI. Serum glucose and albumin were restored to near control levels with treatment. Ghrelin treatment also attenuated WBI-induced intestinal apoptosis by 62% as evidenced by TUNEL staining. The expression of anti-apoptotic cell regulator Bcl-xl was decreased by 38% in the vehicle and restored to 75% of the control with ghrelin treatment. Increased expression of intestinal CD73 and pAkt were observed with ghrelin treatment, indicating protection of the intestinal epithelium after WBI. These results indicate that human ghrelin attenuates intestinal injury and mortality after WBI. Thus, human ghrelin can be developed as a novel mitigator for radiation injury.

## Introduction

The widespread use of nuclear energy in generation of electrical power, and in diagnostic and treatment modalities, has led to greater recognition of the serious and dangerous effects of radiation exposure. Radiation exposure can cause damage in every major organ system [1,2]. Acute radiation syndrome (ARS) is defined as signs and symptoms relating to exposure to radiation. Despite advances in our understanding of acute radiation sickness, its management is mainly supportive. Although efforts aimed at radiation countermeasures were initiated more than half a century ago, there still exists an unmet medical need for an effective therapy in radiation sickness or ARS.

Ghrelin is a 28-amino acid peptide principally produced in endocrine cells of the stomach, termed X/A-like or ghrelin cells, found in the gastric fundus [3–5]. The biological effects of ghrelin are mediated through the growth hormone secretagogue receptor type 1a (GHSR1a, ghrelin receptor), a 7 transmembrane domain Gq protein-coupled receptor. Ghrelin is the only identified endogenous ligand for this receptor. Stimulation of pituitary GHSR1a by ghrelin is reported to induce growth hormone release [6–8]. In addition, a large body of evidence has indicated other physiological functions of ghrelin which are mediated by both central and peripheral ghrelin receptors [9]. Ghrelin has been linked to the regulation of feeding, energy homeostasis, and function in the pituitary, gastrointestinal, cardiovascular, and immune system. The wide distribution of ghrelin receptors suggests multiple paracrine, autocrine, and endocrine roles of ghrelin [10–12].

Previously, we have shown that exogenous administration of ghrelin in animal models of injuries attenuates systemic inflammation [13–16], lung injury [17], gastrointestinal injury [18] and brain injury [19]. We sought to determine whether ghrelin could be protective against whole body irradiation (WBI) and be developed as a radiomitigator for the victims of ARS.

## Materials and Methods

### Experimental animals

Male Sprague-Dawley (SD) rats (250–350 g) purchased from Charles River Laboratories (Wilmington, MA, USA) were used. The rats were housed in a temperature-controlled room on a 12-h light/dark cycle and fed a standard Purina rat chow diet. Animal experimentation was carried out in accordance with the Guide for the Care and Use of Laboratory Animals. This project was approved by the Institutional Animal Care and Use Committee (IACUC) of the Feinstein Institute for Medical Research.

### Animal model of whole body irradiation

Rats were exposed to whole body irradiation (WBI) of 10 Gray (Gy) using a Gammacell 1000 Irradiator (Atomic Energy of Canada Ltd) [radiation source: Cesium-137 (_137_Cs)]. The 10 Gy dose was delivered at a dose rate of 2.5 Gy/min for 4 min. The animals were anesthetized with IP pentobarbital (40 mg/kg BW) prior to irradiation. During radiation, the container rotated continuously in front of the radiation source for even exposure. The Gammacell 1000 is equipped with a cylindrical container that moves inside the machine during irradiation and exits outside after the scheduled radiation time. Rats were anesthetized with pentobarbital to keep them sedated while inside the Gammacell 1000 for irradiation. The animals were then returned to their cages, and food and water were provided.

### Administration of human ghrelin

Rats were exposed to WBI as described above and randomly assigned to control, treatment or vehicle groups. Rats in the treatment group received human ghrelin (20 nmol/rat) subcutaneously once a day for 6 days after WBI. In the vehicle group, human ghrelin was replaced with an equivalent volume of normal saline (NS). Age and weight matched non-irradiated animals were used as controls.

### Survival study

To assess the survival benefits of human ghrelin, animals were exposed to 10 Gy WBI and treated with human ghrelin (20 nmol/rat) subcutaneously once a day (morning) with the first dose given either at 24 h or 48 h after WBI for 6 days and observed for 30 days and the survival rate recorded. The surviving animals beyond 30 days were euthanized. Rats in the survival study were monitored twice daily by experienced lab members and weighed once a day during the 30 day survival period. The net weight change was calculated in percentages. We chose not to administer any long-lasting analgesics agents, such as opioids or non-steroidal anti-inflammatory analgesics, as these are reported to have direct immune and pathophysiological effects. Rats were housed three per cage with environmental enrichments (i.e., red rat huts), and unlimited water and food pellets were provided. The animals were well cared for by the animal care personnel and by the veterinarian at our institution. There are specific criteria for studies that involve death as an endpoint set forth by the IACUC at the Feinstein Institute for Medical Research and agreed upon by our investigators. They include: 1) animals will be euthanized when death can be reasonably predicted; 2) animals approaching a pre-moribund state will be specifically monitored twice daily by an experienced person and written records maintained; 3) animals will be euthanized if they are unable to stand or display agonal breathing, severe muscular atrophy, severe ulceration or uncontrolled bleeding. The specific criteria to identify animals for euthanasia in this study were impaired ambulation, severe bleeding, and chronic diarrhea. None of the animals in either the vehicle group or the treatment group met any of these criteria to be euthanized. The Animal Research: Reporting In Vivo Experiments (ARRIVE) guidelines checklist is shown (S1 ARRIVE Checklist).

### Blood and tissue collection after WBI

Rats exposed to 10 Gy WBI were treated with 20 nmol/rat human ghrelin once a day for 6 days starting at 24 h after radiation. On the 7^th^ day, rats were euthanized, and blood, liver, and the small intestine were removed under sterile conditions. The blood was allowed to clot, and following centrifugation, the serum was transferred into pyrogenic-free sterile tubes, and stored at -80°C until further analysis. Liver (0.25 g) was snap-frozen in liquid nitrogen and stored at -80°C for subsequent bacterial translocation analysis. Ileum (10–15 cm) was harvested for gut permeability studies, jejunum (10 cm) was collected for histopathology, and the remaining small intestine (10 cm) was collected for RNA and protein analyses.

### Plasma ghrelin levels after WBI

Blood samples were collected into a tube containing EDTA and aprotinin at 7 days post WBI. Blood was centrifuged at 2,000 *g* for 10 min at 4°C and plasma collected and stored in -80°C until assay. Ghrelin levels were determined using the Enzyme Immunoassay Kit (Phoenix Pharmaceuticals, Inc., Burlingame, CA) based on manufacturer’s instructions. The levels were calculated from a standard curve ranging 0–100 ng/ml.

### Histopathology

Jejunal segments were fixed in 1:10 buffered formalin and embedded in paraffin. Tissue blocks were sectioned at a thickness of 5 μm, transferred to glass slides, and stained with hematoxylin and eosin (H&E). The sections were observed under 100× magnification using a Nikon Eclipse Ti inverted microscope equipped with the Nikon Digital Sight Camera (Nikon Inc. Melville, NY). Villus height was determined by measuring the distance from the villus-crypt junction to the villus tip, and crypt depth was determined by the distance from villus-crypt junction to basal crypt cells using the Nikon software. For each rat, 100 measurements of villus height and crypt depth were obtained from 5 circumferences. Periodic Acid Schiff (PAS) stained sections were used to quantify the number of goblet cells per villus. For each rat, 60–80 villi were counted for goblet cell quantification. Three rats in each group were used to calculate the mean.

### Gut permeability

Small intestinal mucosal barrier function was assessed by using the ex-vivo isolated everted ileal sac as originally described by Wattanasirichaigoon, *et al* in 1999 [20], used by others [21,22] and recently by us [23]. Briefly, everted gut sacs were prepared in ice-cold modified Krebs-Henseleit bicarbonate buffer (KHBB, pH 7.4). Fluorescein isothiocyanate dextran with a molecular weight of 4000 Da (FD4) was used as a permeability probe. Fluorescence measurements were made with a CYTOFLUOR 2 fluorescence plate reader (PerSeptive Biosystems, Framingham, MA) at an excitation wavelength of 480 nm and an emission wavelength of 520 nm. Gut permeability was expressed as the mucosal-to-serosal clearance of FD4 as described in detail by Wattanasirichaigoon *et al* [20].

### Serum endotoxin levels

Bacterial endotoxin levels in the serum were determined by the endpoint chromogenic Limulus Amebocyte Lysate (LAL) assay (QC-1000, Lonza, Walkervilles, MD USA) and expressed in EU/ml. Samples were analyzed according to manufacturer’s instruction. Endotoxin levels which are ≥ 0.1 EU/ml can be measured using this assay.

### DNA isolation and total bacteria quantification

DNA was isolated from liver homogenates using DNeasy blood & tissue kits (QIAGEN Gmbh, D-4072 Hilden). Frozen liver tissue (0.25 mg) was powdered and DNA was isolated. Bacterial quantification was performed using 16S rRNA gene-targeted primer, forward: 5’-AAC GCG AAG AAC CTT AC-3’ and reverse: 5’-CGG TGT GTA CAA GAC CC-3’. Amplification of DNA by real time PCR was performed with 7300-qPCR using 96-well PCR plates. The PCR reaction was performed in a total volume of 25 μl containing 100 ng template DNA, 0.1 μM of each primer and 12 μl 2X SYBR Green PCR Master Mix (Applied Biosystems, Foster City, CA). The PCR conditions for total bacterial quantification were 95°C for 1 min and 40 cycles of 95°C for 20 sec and 60°C for 1 min. A melting curve analysis was made after amplification. Serially diluted genomic DNA of bacterial isolates was used as real time PCR control for bacterial quantification. A standard curve was generated from the bacterial DNA control and we consistently detected between 100 ng to 0.1 pg *E*. *coli* DNA. Bacterial counts were expressed as ng/mg liver tissue.

### Serum glucose and albumin levels

Serum levels of glucose and albumin were determined using Liquid Glucose Hexokinase Reagent Set and Albumin Reagent Set (Pointe Scientific, Inc.) respectively, according to manufacturer’s instructions.

### TUNEL staining

The presence of apoptotic cells in the gut sections were assessed on the 7^th^ day following WBI using a terminal deoxynucleotide transferase dUTP nick-end labeling (TUNEL) staining kit (Roche Diagnostics, Indianapolis, IN). The negative control was performed by incubating slides in the mixture containing only deoxynucleotidyl transferase. TUNEL-positive cells in crypt were counted in 10 fields/section under a fluorescence microscope (200×), and the number of cells/crypt are shown.

### Western blot analysis

Small intestine (100 mg) was lysed and homogenized in 1 ml lysis buffer (10 mM TBS, 1 mM EDTA, 1 mM EGTA, 2 mM sodium orthovanadate, 0.2 mM PMSF, 2 μg⁄ml leupeptin, 2 μg ⁄ml aprotinin, and 1% Triton X-100) for 30 min on ice and cleared by centrifugation at 12,000 *g* for 15 min at 4°C. Protein (80 μg) was fractionated on a 4 to 12% Bis-Tris gel and transferred to a 0.2-μm nitrocellulose membrane. Nitrocellulose blots were blocked by incubation in TBST (10 mM Tris-HCl, pH 7.5, 150 mM NaCl, and 0.1%Tween 20) containing 5% milk for 1 h at room temperature. Western blotting was performed using the following primary antibodies at 1:1,000 dilutions: anti-Bcl-xl antibody, anti-Bcl-2 polyclonal antibody (N-19), anti-Bax antibody, anti-VEGF antibody (Santa Cruz Biotechnology), and anti-pAkt antibody (Cell Signaling). After overnight incubation with the primary antibodies at 4˚C, the membranes were washed with TBST. Immunoreactive bands were detected using HRP-linked secondary antibody (Southern Biotech, Birmingham, AL) and the Enhanced Chemiluminescence (ECL) Western blot detection kit (Amersham, Piscataway, NJ). The immunoblots were exposed to X-ray film and analyzed with the NIH Image J analysis system. Mouse anti-β-actin monoclonal antibody (1:10,000; Sigma) was used as a loading control.

### Total RNA extraction and real time PCR

Total RNA was extracted from the jejunum by Tri-Reagent (Molecular Research Center, Cincinnati, OH). RNA (4 μg) from each sample was reverse-transcribed in a 20 μl reaction volume containing 10× PCR buffer, 5 mM MgCl_2_, 1 mM dNTP, 20 U RNase inhibitor, and 50 U reverse transcription. 2.5 mM oligod(T_16_)primer (Invitrogen, Grand Island, NY). The reverse transcription reaction solution was incubated at 42°C for 1 h, followed by heating at 95°C for 5 min; 2 μl cDNA was amplified with 0.15 μM each of 3′ and 5′ primers specific for rat ICAM-1 and CD73. Rat glyceraldehyde 3-phosphate dehydrogenase (GAPDH) was used as the housekeeping gene. The primer sequences are the following: ICAM-1 forward: 5’-CGA GTG GAC ACA ACT GGA AG-3’ and reverse: 5’- CGC TCT GGG AAC GAA TAC AC-3’; CD73 forward: 5’-CAC AGG AAA TCC ACC TTC CAA-3’and reverse: 5’-ATC GTC AGA GGT GAC TAT GAA TGG-3’; GAPDH forward: 5’-ATG ACT CTA CCC ACG GCA AG- 3’ and reverse: 5’-CTG GAA GAT GGT GAT GGG TT-3’. Each cycle consisted of 30 sec at 94°C, 30 sec at 60°C, and 45 sec at 72°C.

### Statistical analysis

Based on sample size calculation by ANOVA, if the difference between groups is 40–45% with a standard deviation of 25%, the minimum number of animals required for the short term studies (i.e., post WBI day 7) is 7 animals per group. The majority of experiments were conducted with 6–7 rats per group. Based on the assumption that the differences between groups using sample size estimation will be around 35% and standard deviation represents 25% of the mean, 27 animals per group were required for the survival studies. Since we expected the survival to be greater in the treated animals than non-treated ones, 25 animals per group were estimated for the survival study. We achieved statistical significance with a sample size of n = 16/group for 24 h post treatment and n = 23/group for 48 h post treatment. All data are expressed as mean ± SE and analyzed by one way analysis of variance (ANOVA) and compared using Student Newman Keul’s test for multiple comparisons. Student’s *t*-test was used for two group analysis. The survival curves were plotted using the Kaplan-Meier analysis and the curves were subjected to the Log Rank test. The differences in values were considered significant if P < 0.05.

## Results

### Ghrelin improved survival after whole body irradiation (WBI)

**Fig 1:**
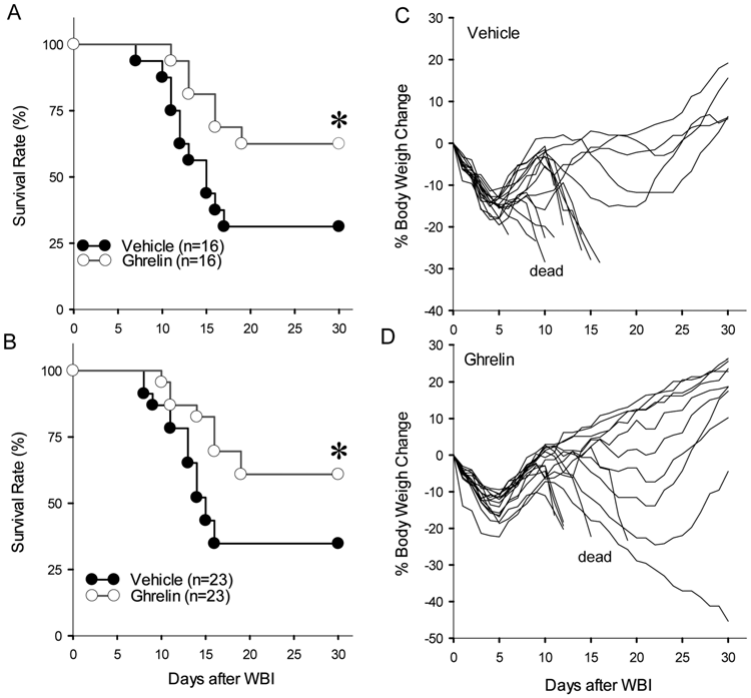
Human ghrelin improves survival after WBI. Male Sprague-Dawley rats were exposed to 10 Gy WBI and treated with vehicle or human ghrelin (20 nmol/rat) for 6 days starting at 24 h (A) or 48 h (B) and observed for 30 days. The survival rate was estimated by the Kaplan-Meir method and compared by Log Rank test. *P = 0.04 vs. Vehicle. The percent body weight change was calculated for each animal from Vehicle (C) and human ghrelin treatment starting at 24 h (D) and plotted. Those animals in both groups that were dead as designated as such.

We previously determined a 10 Gray (Gy) radiation dose as the LD_70/21_ of WBI for our experimental cohort (adult male SD rats) using the Gammacell 1000 irradiator [24]. To evaluate ghrelin’s effect on WBI, 10 Gy WBI rats were treated with a vehicle or human ghrelin (20 nmol/rat) once a day for 6 days starting at either 24 h or 48 h after WBI. As expected, we achieved the LD_70/21_ in our vehicle group and the survival rate remained 30% during the 30 day monitoring period of the study (LD_70/30_). When WBI rats were treated with human ghrelin starting at 24 h after WBI, the survival rate was 63% which was significantly higher than that of the vehicle group (Fig. 1A). When treatment was initiated at 48 h after WBI, the survival rate remained significantly high at 61% (Fig. 1B). This survival benefit was evident in the percent body weight change where the majority of rats in the vehicle group (Fig. 1C) continued to lose weight and died whereas those in the treatment group showed a consistent increase in body weight and survived (Fig. 1D).

### Plasma ghrelin levels decreased after WBI

**Fig 2:**
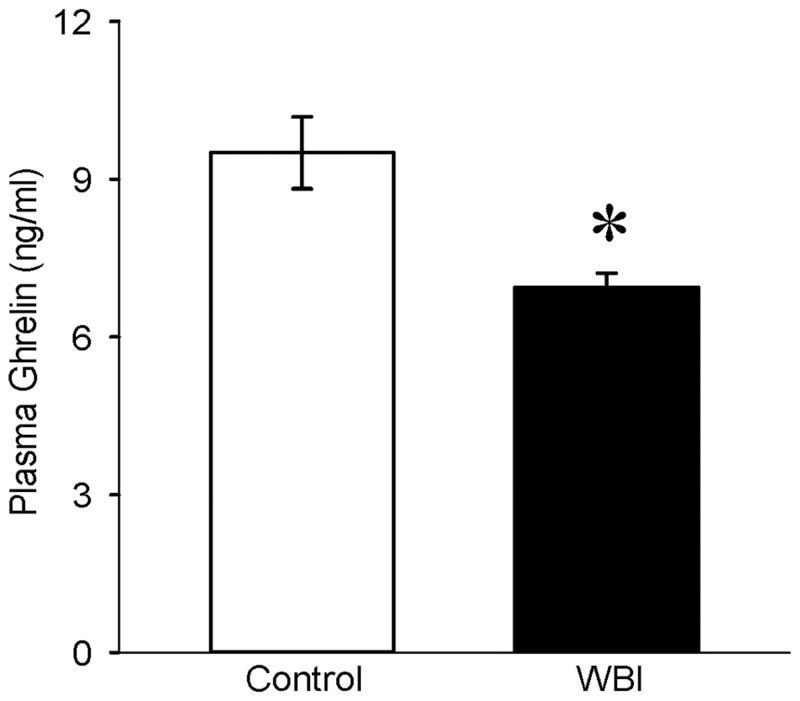
Plasma ghrelin levels after WBI. Ghrelin levels from plasma of non-irradiated (Control) and 10 Gy WBI were measured using an Enzyme Immunoassay Kit. Ghrelin levels (ng/ml) were calculated against a standard curve ranging from 0 to 100 ng/ml. Data are presented as mean ± SE (n = 6–7) and compared with Student’s *t*-test. *P = 0.008 vs. Control.

To evaluate whether circulating levels of ghrelin are altered after WBI, ghrelin in plasma samples from control and WBI animals were measured. At 7 days post WBI, ghrelin levels were significantly decreased by 27% which raised the possibility of treating WBI with ghrelin (Fig. 2).

### Ghrelin improved intestinal integrity and morphology after WBI

**Fig 3:**
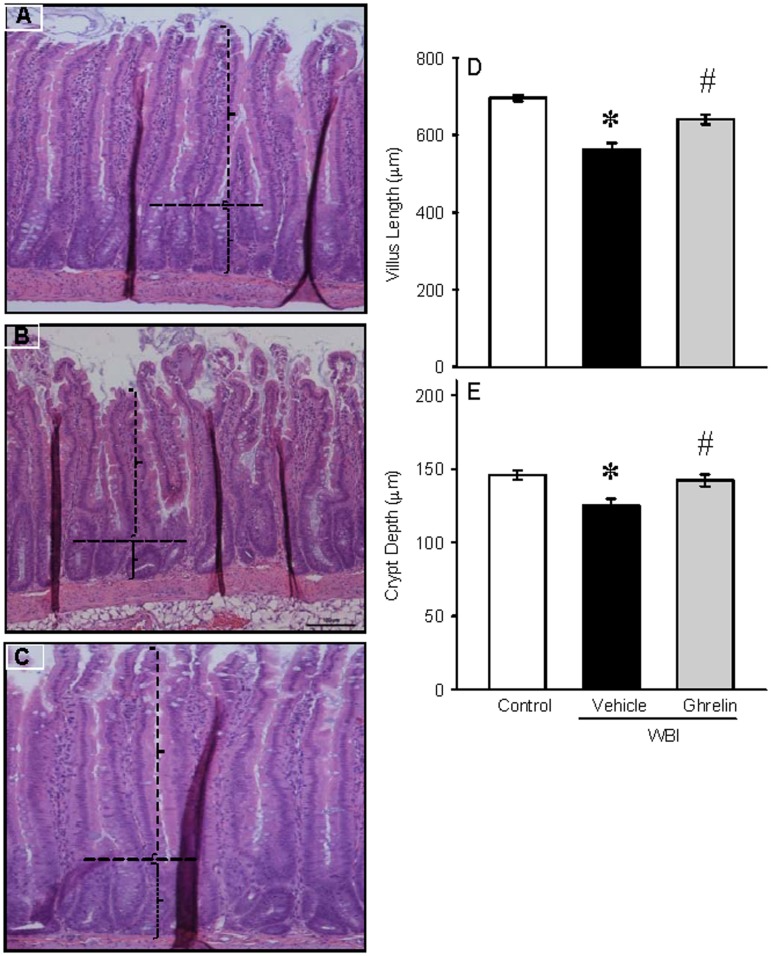
Histology of rat jejunum after WBI. Histological sections of the rat jejunum from Control (A), Vehicle (B) and human ghrelin (C) were stained with hematoxylin and eosin stain (100 × magnification). Villus length (D) and crypt depth (E) were measured using Nikon software and indicated by vertical and horizontal dotted lines, respectively. Data are presented as mean ± SE (n = 3) and compared by Student Neuman Keul’s test by ANOVA. *P<0.001 vs. Control; ^#^P<0.003 vs. Vehicle.

**Fig 4:**
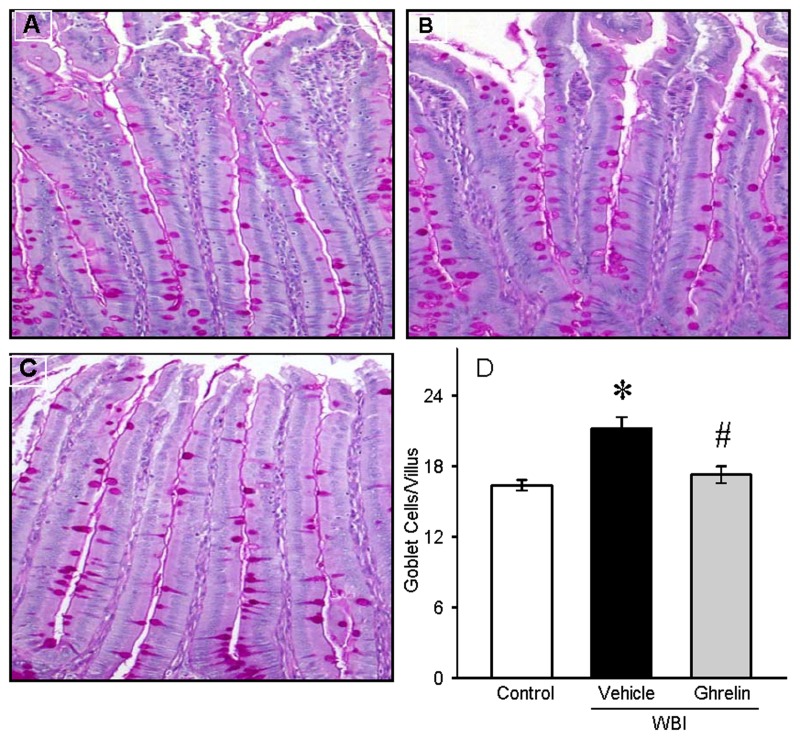
Periodic Acid Schiff (PAS) staining of rat jejunum after WBI. Histological sections from Control (A), Vehicle (B) and human ghrelin (C) were stained with PAS (200 × magnification). Goblet cells/villus (D) was counted using Nikon software. Data are presented as mean ± SE (n = 3) and compared by Student Neuman Keul’s test by ANOVA. *P<0.001 vs. Control; ^#^P<0.001 vs. Vehicle.

To determine the effect of human ghrelin on gut morphology, gut sections from control, vehicle, and ghrelin treated groups were stained with H & E and examined under the light microscope (Fig. 3). Villus length and crypt depth were assessed. On day 7 after WBI, there was still denudation and altered morphology of the villi and crypts. Treatment with human ghrelin for 6 days starting at 24 h after WBI improved the intestinal integrity and morphology. As shown in Fig. 3D, the villus length in the vehicle group was significantly reduced by 19% while the treatment group had only a 2% reduction from the control. Likewise, the crypt depth was also significantly decreased by 14% in the vehicle group while the treatment group had only a 2.8% reduction from the control (Fig. 3E). Increase in goblet cell number is an indicator of gut injury. The sections were stained with PAS, the goblet cell stain, and examined under light microscopy (Fig. 4). The number of stained goblet cells in the control, vehicle and treatment groups was assessed. As shown in Fig. 4D, goblet cell number/villus in the vehicle group was significantly increased by 29% while the treatment had only a 5% increase from the control.

### Ghrelin attenuated gut permeability, serum endotoxin, and bacterial translocation after WBI

**Fig 5:**
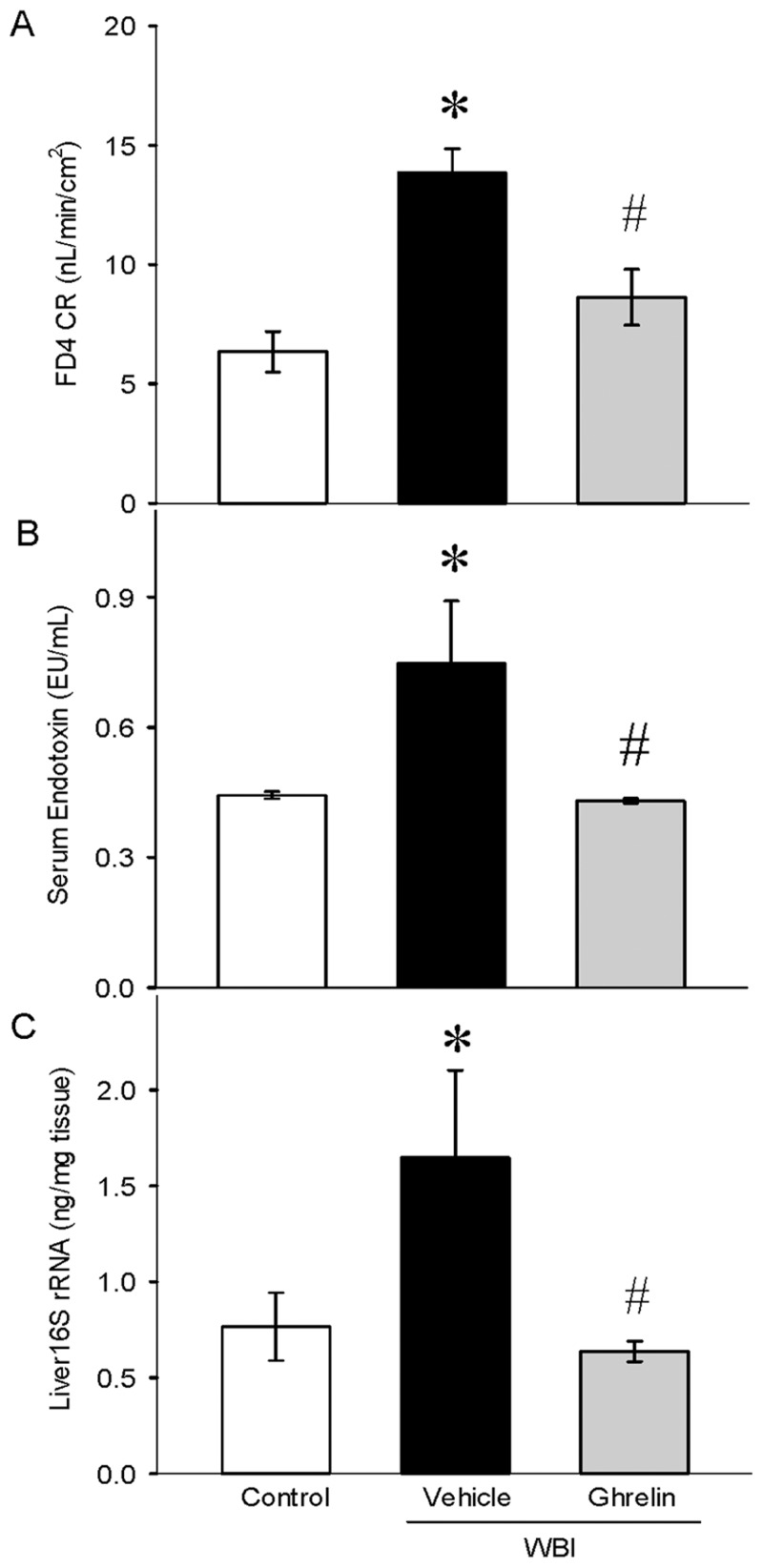
Gut permeability, serum endotoxin levels, and bacterial translocation after WBI. Gut permeability of the rat ileum (A) was assessed. Data are presented as mean ± SE (n = 5–6) and compared by Student Neuman Keul’s test by ANOVA. *P<0.001 vs. Control; ^#^P<0.004 vs. Vehicle. Serum endotoxin levels (B) were determined by the endpoint chromogenic Limulus Amebocyte Lysate (LAL) assay and expressed in EU/ml. *P<0.025 vs. Control; ^#^P<0.05 vs. Vehicle. Liver 16S rRNA was performed using real time PCR. Bacterial DNA was used as standards and the counts are expressed as ng/mg tissue. *P<0.04 vs. Control; ^#^P<0.04 vs. Vehicle.

Small intestinal mucosal barrier function was assessed by using the ex-vivo isolated everted ileal sac. On day 7 after WBI, gut permeability increased by 118% in the vehicle animals and was reduced significantly in the treatment group by 38% compared to the vehicle, restored to near control levels (Fig. 5A). Increased gut permeability allows passage of bacteria into the circulation and subsequent translocation to tissues. In the vehicle-treated animals, serum endotoxin levels were increased by 68% while the treatment animals showed a significant decrease by 42% (Fig. 5B). Likewise, 16S rRNA gene, a measure of bacterial counts, was significantly elevated in the liver tissue by 115% and reduced by 61% from Vehicle after ghrelin treatment (Fig. 5C).

### Ghrelin restored serum glucose and albumin levels after WBI

**Fig 6:**
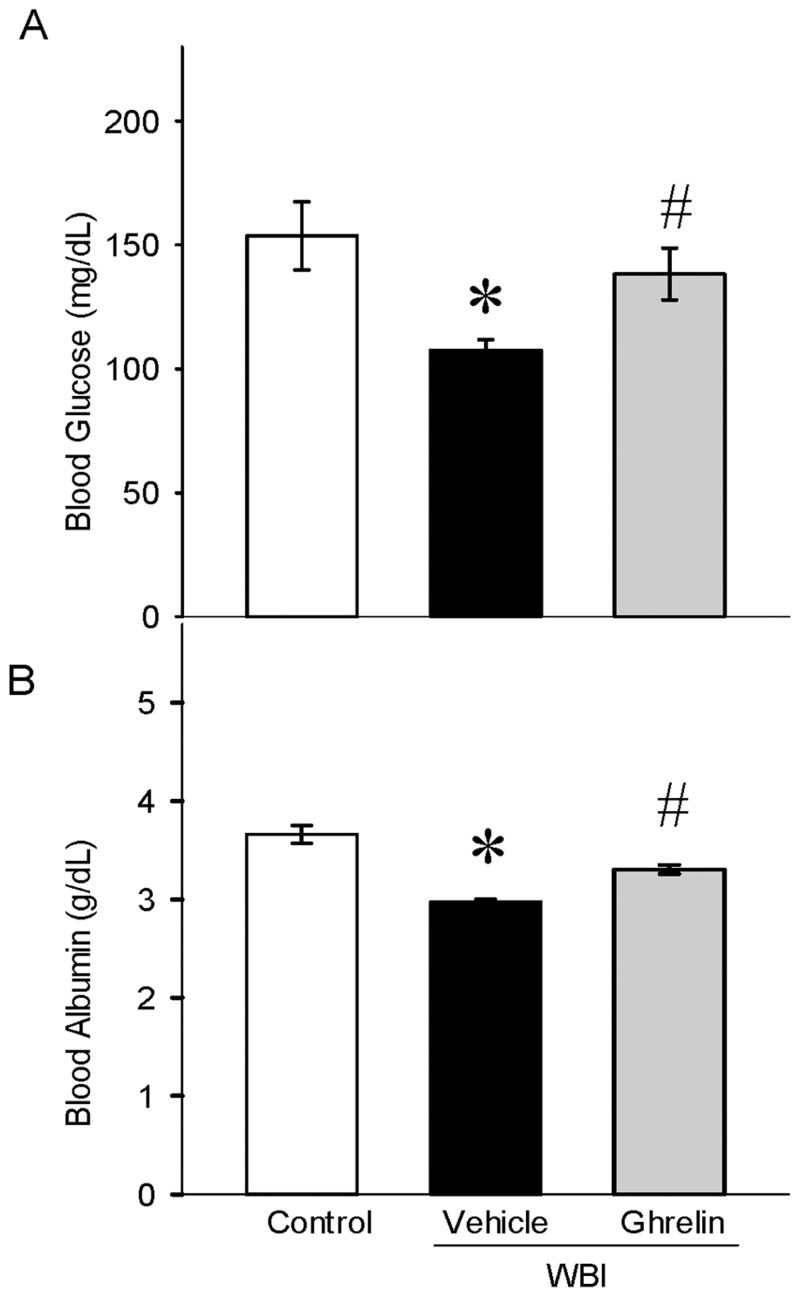
Serum glucose and albumin levels after WBI. Serum glucose (A) and albumin (B) were measured using Liquid Glucose Hexokinase Reagent Set and Albumin Reagent Set, respectively. Data are presented as mean ± SE (n-6–7) and compared by Student Neuman Keul’s test by ANOVA. *P<0.01 vs. Control; ^#^P<0.03 vs. Vehicle.

Blood glucose and albumin has been thought to decrease after radiation injury. On day 7 after WBI, the serum glucose was significantly reduced by 30% and restored to near control levels with ghrelin treatment (Fig. 6A). Likewise, serum albumin levels were decreased significantly by 19% and restored to control values with ghrelin treatment (Fig. 6B).

### Ghrelin attenuated intestinal apoptosis after WBI

**Fig 7:**
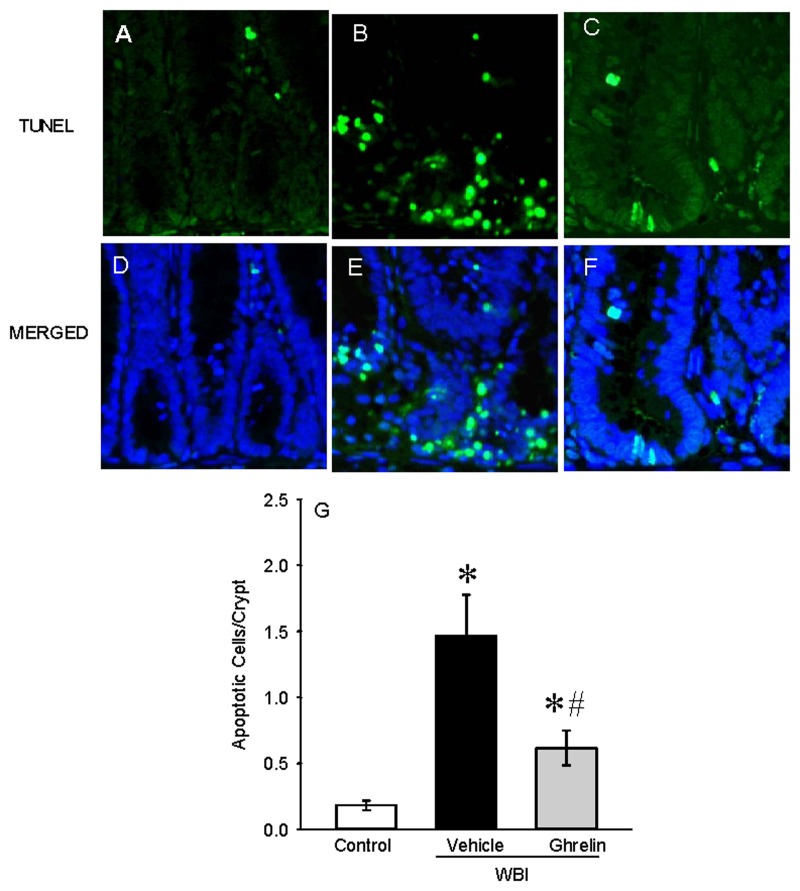
Intestinal TUNEL staining after WBI. Jejunal sections from Control (A, D), Vehicle (B, E) and human ghrelin treatment (C, F) were stained with terminal deoxynucleotide transferase dUTP nick-end labeling (TUNEL) staining kit. The sections were counterstained with DAPI and images were merged with the TUNEL stain (MERGED). TUNEL positive cells/crypt (D) was counted. Data are presented as mean ± SE (n = 3) and compared by Student Neuman Keul’s test by ANOVA. *P<0.001 vs. Control; ^#^P<0.001 vs. Vehicle.

**Fig 8:**
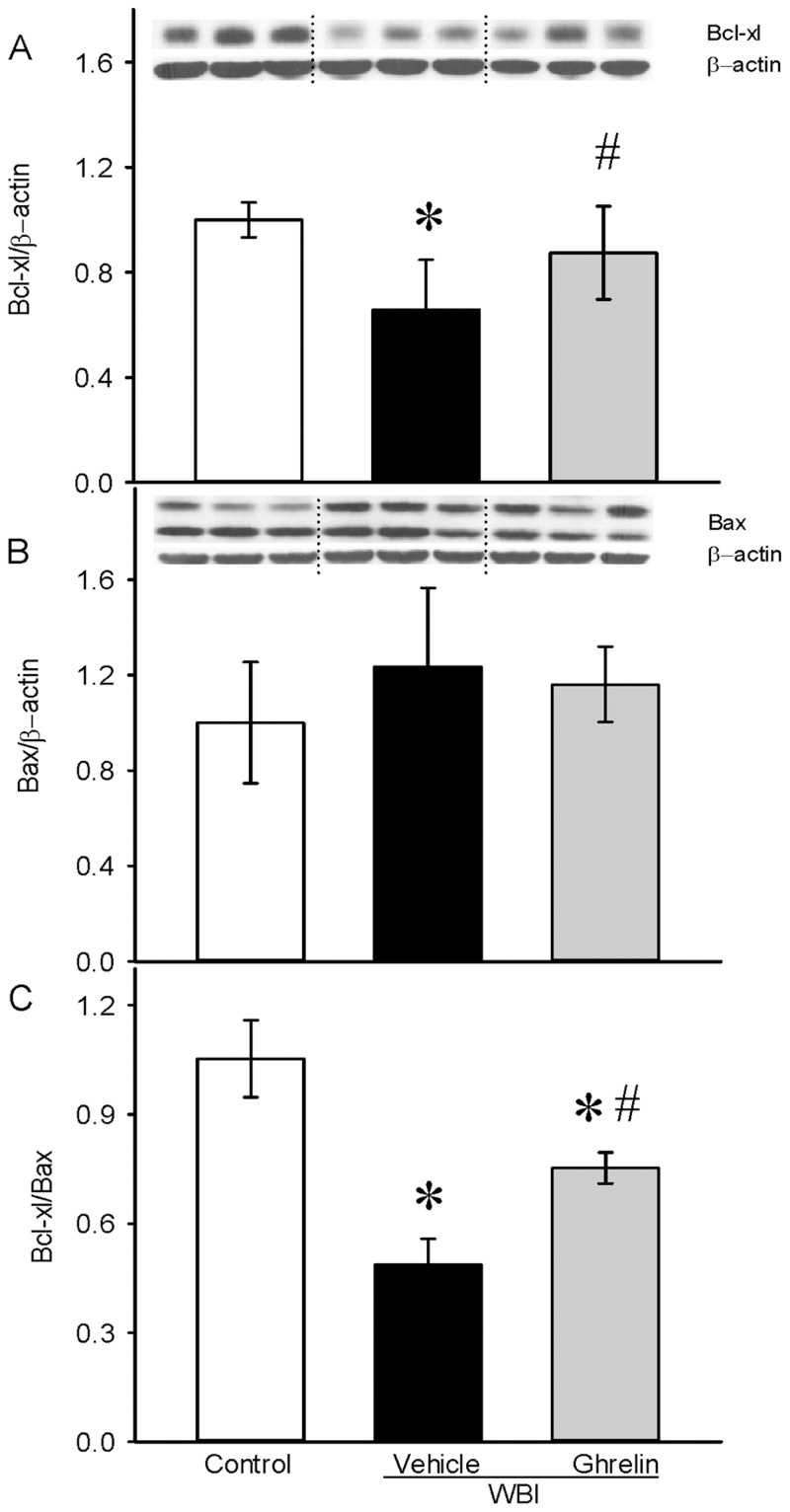
Bcl-xl and Bax protein levels after WBI. Protein lysates from Jejunal segments were examined by Western blotting for Bcl-xl (A) and Bax (B) protein levels. Bcl-xl/Bax ratio (C) was calculated and plotted. Data are presented as mean ± SE (n = 6–7) and compared by Student Neuman Keul’s test by ANOVA. *P<0.001 vs. Control; ^#^P<0.011 vs. Vehicle.

Intestinal injury leading to crypt cell death is commonly observed in high dose radiation. Apoptotic cell death in various organs is known to be prevalent in animal models of injuries. To determine ghrelin’s effect on intestinal crypt apoptosis, histological sections from the jejunum of control, vehicle and ghrelin treated animals were stained with TUNEL (Fig. 7). On day 7 after WBI, the number of apoptotic cells per crypt increased by 638% in the vehicle group from control values and treatment with ghrelin reduced these numbers by 62% (Fig. 7D). In addition, protein expression levels of Bcl-xl (anti-apoptotic) and Bax (pro-apoptotic) markers were assessed in the jejunal samples. A significant 34% reduction of the Bcl-xl protein levels was observed on day 7 after WBI whereas these levels were restored to 75% of the control with ghrelin treatment (Fig. 8A). The Bax protein was slightly elevated in the vehicle group but remained the same as control after ghrelin treatment (Fig. 8B). Interestingly, the vehicle group showed a significant 54% reduction of the Bcl-xl/Bax ratio, whereas with ghrelin treatment, this ratio is significantly increased 55% from the vehicle and approximates control levels (Fig. 8C).

### Ghrelin attenuated leukocyte trafficking and protected the intestinal epithelium after WBI

**Fig 9:**
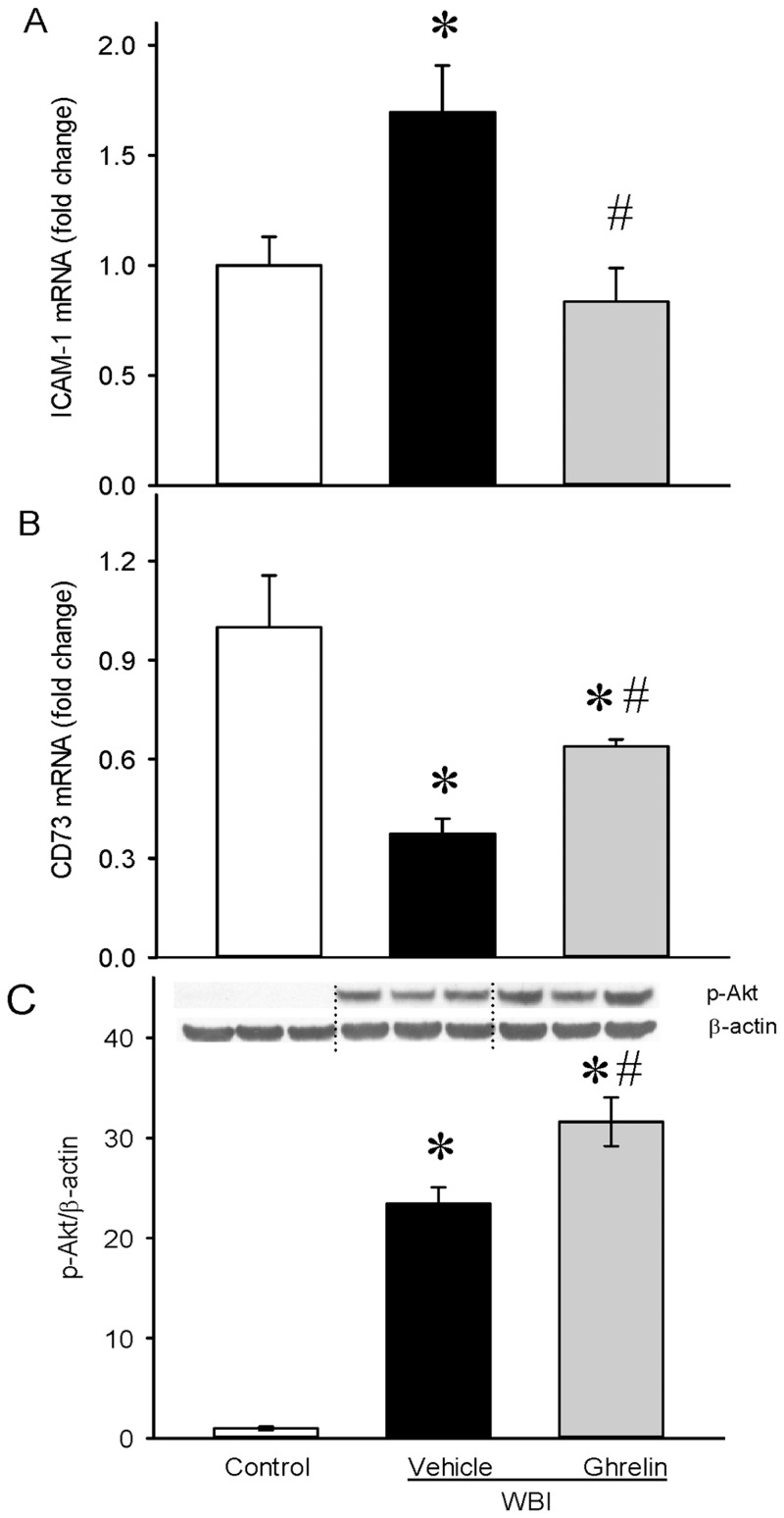
Leukocyte trafficking and intestinal epithelial cell survival after WBI. Total RNA from jejunal segments were examined for ICAM-1 (A), CD73 (B) mRNA expressions by real time PCR. Protein lysates from jejunal segments were examined for pAkt expression (C) by Western blotting. Data are presented as mean ± SE (n = 6–7) and compared by Student Neuman Keul’s test by ANOVA. *P<0.05 vs. Control; ^#^P<0.05 vs. Vehicle.

Break down of the mucosal barrier during radiation injury exposes subepithelial tissues to the contents of the intestinal lumen. Endothelial cell barrier dysfunctions cause leukocyte trafficking to tissue. Intercellular adhesion molecule-1 (ICAM-1) is an endothelial and leukocyte associated transmembrane protein in stabilizing cell-cell interactions [25]. As shown in Fig. 9A, ICAM-1 mRNA expression was increased by 69% and ghrelin treatment showed a significant 51% inhibition from the vehicle levels. Tissue-Resident Ecto-5’Nucleotidase (CD73) prevents leukocyte trafficking by tightening the epithelial barrier [26,27]. As shown in Fig. 9B, CD73 mRNA expression was significantly decreased by 62% in the vehicle, and ghrelin treatment restored its expression by 70%, suggesting the protection of the intestinal epithelial barrier by ghrelin. Phospatidylinositol 3-kinase/Akt pathway activation is essential for intestinal epithelial cell proliferation. As shown in Fig. 9C, intestinal Akt is activated in response to radiation and ghrelin treatment further significantly increased its expression indicating the role of cell proliferation and survival by ghrelin after WBI.

## Discussion

Ionizing radiation is utilized in radiotherapy and nuclear imaging for diagnosis as well as in the generation of electrical power. The widespread use of radiation has yielded equal concern regarding its danger to the general public. While nuclear accidents are seldom occurrences, the events at Chernobyl and Fukushima as well as the threat of nuclear terrorism further necessitates mitigation strategies. The treatment of radiation victims has been a major focus, with research concentrated on therapeutic agents which are known as radiomitigators. Granulocyte colony stimulating factor (G-CSF) based on its efficacy has obtained the FDA approval as the Emergency Use Investigational New Drug status for acute radiation syndrome [28]. One major limitation of G-CSF is the timing of administration after irradiation. Studies have shown that efficacy of G-CSF administration is referred to as “the earlier the better,” with 2 h post-irradiation being optimal [29]. Therapy using combinations of G-CSF with other agents such as interleukin-3 receptor agonist [30], amifostine [31] and adenosine receptor agonist [31,32] were postulated. As such, there is still an unmet medical need that exists for an effective radiomitigator.

In the present study, we demonstrated that human ghrelin, a stomach peptide given starting at either 24 h or 48 h post WBI and continued for 6 days, significantly improved survival rate in rats during the 30 day observation period. The survival rate positively correlated with increased body weight in the ghrelin treated animals and was similar to those animals in the vehicle group that survived the insult. At 7 days post WBI, histological appearance was altered as evidenced by the significant decrease in villus length and crypt depth. Ghrelin treatment starting at 24 h and continuing for 6 days after the irradiation improved the histology and attenuated the goblet cell number/villus indicating restoration of the intestinal mucosa. At 7 days post WBI, gut permeability, serum endotoxin levels, and bacterial translocation to the liver were augmented, indicating increased susceptibility to infection. Ghrelin treatment significantly reduced these parameters demonstrating its ability to attenuate radiation-induced inflammation and susceptibility to infection. While it is well accepted that DNA breaks occur after radiation exposure, very little is known about the role of apoptosis of the gastrointestinal tract in radiation injury. We show that at 7 days post WBI, a significant increase in TUNEL positive cells are seen in the intestine and ghrelin treatment attenuated the number of these cells and brought down to near control values. In addition, ghrelin treatment attenuated endothelial cell activation, and improved intestinal epithelial cell integrity and cell survival. These results collectively demonstrate the protective effect of ghrelin after radiation exposure, and it underscores its ability to serve as a radiation mitigator.

We first examined the effect of human ghrelin on survival after WBI. A prior pilot study was conducted with 7.5 Gy and 10 Gy WBI to establish the LD_50_ model for our experimental cohort. At 7.5 Gy, we only observed a 10% mortality and at 10 Gy, the mortality significantly increased to 70% in the 21-day observation period. Based on this, we chose to expose the rats to 10 Gy WBI to determine the effect of human ghrelin in radiation injury. For an agent to be considered as a radiomitigator, the compound should be protective if administered 24 h or later after WBI. Based on this criterium, the treatment with human ghrelin was initiated at either 24 h or 48 h after WBI. When treatment was initiated at 24 h or 48 h after WBI, the survival rate was 63% and 61% respectively, suggesting that human ghrelin could be developed as a radiomitigator.

In addition to the survival study, we examined the effect of human ghrelin treatment initiated at 24 h after 10 Gy WBI on gastrointestinal integrity. While intestinal mucosal damage can be achieved by exposure to 8 or even 6 Gy [33], the 10 Gy radiation exposure chosen for the current study was to achieve mortality rather than to just to induce intestinal injury. At 10 Gy WBI, we observed mortality only after day 8 following WBI and therefore, day 7 post WBI was chosen to examine gastrointestinal integrity. At 7 days post WBI (i.e., 24 h after the termination of treatment), the small intestine was examined for intestinal integrity as measured by histopathology (villus height, crypt depth and goblet cell number per villus) and gut permeability. Ghrelin treatment improved the histological appearance and restored the intestinal mucosa. Gastrointestinal damage leads to the leakage of intestinal contents such as bacteria and bacterial products. Ghrelin treatment reduced gut permeability and prevented leakage of endotoxin to the circulation and reduced bacterial translocation to the liver. These data suggest that ghrelin treatment attenuated radiation induced intestinal damage. The damage to the intestine leads to crypt death and apoptotic cell death in various organs is known to be prevalent in animal models of injuries. Ghrelin treatment significantly attenuated intestinal apoptosis following radiation. Break down of the mucosal barrier during radiation injury exposes subepithelial tissues to the contents of the intestinal lumen. Endothelial cell barrier dysfunctions cause leukocyte trafficking to tissue. Intercellular adhesion molecule-1 (ICAM-1) is an endothelial and leukocyte associated transmembrane protein in stabilizing cell-cell interactions. Tissue-Resident Ecto-5’Nucleotidase (CD73) prevents leukocyte trafficking by tightening the epithelial barrier [26,27]. Ghrelin treatment decreased ICAM-1 and restored CD73 expression suggesting the protection of the intestinal epithelial barrier. Phospatidylinositol 3-kinase/Akt pathway activation is essential for intestinal epithelial cell proliferation. Ghrelin treatment increased Akt expression indicating increased cell proliferation of the epithelial cells in the intestine.

The biological effects of ghrelin are mediated through GHSR. It has been reported GHSR is present in afferent neurons of the nodose ganglion suggesting that ghrelin signals are transmitted to the brain by vagal afferent nerves [34]. Also, central administration of ghrelin stimulates the vagus nerve in anesthetized rats [35]. In fact, we have shown that ghrelin’s protective effect in sepsis and other injury conditions were mediated by the vagus nerve [13,19]. It is possible ghrelin’s beneficial effect in radiation injury could be mediated by the vagus nerve. Further studies are warranted for such a conclusion.

Our studies show in a WBI model of LD_70/30_ in SD rats, human ghrelin given as late as 48 h after WBI improved survival rate and that ghrelin treatment started at 24 h after WBI improved histological appearance of the intestine, reduced gut permeability, serum endotoxin levels and bacterial translocation to the liver. Ghrelin treatment also attenuated WBI-induced intestinal apoptosis and preserved intestinal integrity. Therefore, ghrelin is protective against radiation injury and mortality and these data suggest that human ghrelin can be developed as a radiomitigator.

## Supporting Information

S1 ARRIVE Checklist (PDF)
